# Excitation–Contraction Coupling in the Goldfish (*Carassius auratus*) Intact Heart

**DOI:** 10.3389/fphys.2020.01103

**Published:** 2020-09-11

**Authors:** Maedeh Bazmi, Ariel L. Escobar

**Affiliations:** ^1^Quantitative Systems Biology Program, School of Natural Sciences, University of California, Merced, Merced, CA, United States; ^2^Department of Bioengineering, School of Engineering, University of California, Merced, Merced, CA, United States

**Keywords:** whole heart, excitation–contraction coupling, goldfish heart, pulsed local field fluorescence microscopy, electrophysiology

## Abstract

Cardiac physiology of fish models is an emerging field given the ease of genome editing and the development of transgenic models. Several studies have described the cardiac properties of zebrafish (*Denio rerio*). The goldfish (*Carassius auratus*) belongs to the same family as the zebrafish and has emerged as an alternative model with which to study cardiac function. Here, we propose to acutely study electrophysiological and systolic Ca^2+^ signaling in intact goldfish hearts. We assessed the Ca^2+^ dynamics and the electrophysiological cardiac function of goldfish, zebrafish, and mice models, using pulsed local field fluorescence microscopy, intracellular microelectrodes, and flash photolysis in perfused hearts. We observed goldfish ventricular action potentials (APs) and Ca^2+^ transients to be significantly longer when compared to the zebrafish. The action potential half duration at 50% (APD_50_) of goldfish was 370.38 ± 8.8 ms long, and in the zebrafish they were observed to be only 83.9 ± 9.4 ms. Additionally, the half duration of the Ca^2+^ transients was also longer for goldfish (402.1 ± 4.4 ms) compared to the zebrafish (99.1 ± 2.7 ms). Also, blocking of the L-type Ca^2+^ channels with nifedipine revealed this current has a major role in defining the amplitude and the duration of goldfish Ca^2+^ transients. Interestingly, nifedipine flash photolysis experiments in the intact heart identified whether or not the decrease in the amplitude of Ca^2+^ transients was due to shorter APs. Moreover, an increase in temperature and heart rate had a strong shortening effect on the AP and Ca^2+^ transients of goldfish hearts. Furthermore, ryanodine (Ry) and thapsigargin (Tg) significantly reduced the amplitude of the Ca^2+^ transients, induced a prolongation in the APs, and altogether exhibited the degree to which the Ca^2+^ release from the sarcoplasmic reticulum contributed to the Ca^2+^ transients. We conclude that the electrophysiological properties and Ca^2+^ signaling in intact goldfish hearts strongly resembles the endocardial layer of larger mammals.

## Introduction

In the last 10 years, fish hearts have become a very popular model for studying heart function (Nemtsas et al., 2010; Huttner et al., 2013; Ravens, 2018; van Opbergen et al., 2018b; Zhang et al., 2018). In developmental studies (Novak et al., 2006; Chi et al., 2010; Chablais and Jazwinska, 2012; Ramachandran et al., 2013; Ding et al., 2017), the zebrafish (*Danio rerio*) has been the model of choice not only for the possibility of performing transgenesis (Chopra et al., 2010; Huttner et al., 2013; Konantz and Antos, 2014; Serbanovic-Canic et al., 2017), but also for studying cardiac physiology of larger mammals, including humans (Nemtsas et al., 2010; Ravens, 2018; van Opbergen et al., 2018b). Despite the numerous advantages of using the zebrafish model as an experimental approach to study cardiac function, there are some disadvantages of this model as well. Disadvantages of the zebrafish model include a higher heart rate (Lee et al., 2016) and a shorter action potential (AP) duration (Lin et al., 2015) compared to that of goldfish and a smaller fraction of Ca^2+^-induced Ca^2+^ release (Bovo et al., 2013) from the sarcoplasmic reticulum (SR) when compared to mammals. Furthermore, the small size of the zebrafish heart makes it difficult to perform perfused intact heart measurements. Indeed, although it is possible to cannulate the *bulbus arteriosus* of the zebrafish heart, it is difficult to effectively change the perfusate quickly enough to perform pharmacological experiments. This is due to the low perfusion rate the zebrafish heart needs to maintain good hemodynamic and mechanical conditions. Last, the ventricular wall of the zebrafish heart is not thick enough (Hu et al., 2001) to consistently perform flash photolysis experiments at an intact heart level without undergoing considerable damage.

Despite the frequent use of fish as a cardiovascular model to study human hearts (Nemtsas et al., 2010), much of our knowledge is still limited. In this paper, we propose the use of the goldfish heart as an alternative novel experimental model to study excitation–contraction coupling. Goldfish (*Carassius auratus*) belong to the same family as zebrafish (Málaga-Trillo et al., 2002; Kon et al., 2020), presenting many physiological similarities. Goldfish hearts are similar to zebrafish hearts, presenting similar advantageous characteristics, such as the possibility of being used as an embryological model (Grivas et al., 2014), being altered via transgenesis using CRISPR/Cas9 (Yin et al., 2018), or being used in electrophysiological and Ca^2+^ signaling experiments (Chen et al., 2005; Leo et al., 2019).

Though fish models have been extensively used in the past to gain a better understanding of mammals, it is important to note that there are some cardiac structural differences between mammals and fish. Perhaps the most important features are the absence of the t-tubule system and the smaller size of the fish ventricular myocytes (Vornanen et al., 2002; van Opbergen et al., 2018b). Independently of these structural distinctions, in this paper, we found an encouraging fact; in goldfish, a significant fraction of Ca^2+^ is released from the SR.

To evaluate the main mechanisms of the goldfish heart function during the cardiac cycle, we designed and performed experiments to (i) evaluate the dependency of the AP and Ca^2+^ transients on heart rate and temperature, (ii) assess the role of L-type Ca^2+^ currents in the excitability of the myocytes as well as its contribution to Ca^2+^ transients, and (iii) weigh the contribution of SR Ca^2+^ release to Ca^2+^ transients and AP repolarization. These goals were tackled using a combination of pulsed local field fluorescence microscopy (Mejía-Alvarez et al., 2003; Escobar et al., 2004, 2006; Aguilar-Sanchez et al., 2017), sharp microelectrode intracellular recordings (Ferreiro et al., 2012; López Alarcón et al., 2019; Aguilar-Sanchez et al., 2019), and flash photolysis (Escobar et al., 2012; Ramos-Franco et al., 2016; López Alarcón et al., 2019; Aguilar-Sanchez et al., 2019). The results presented here demonstrate how the duration and kinetics of the goldfish epicardial AP are compatible with endocardial APs of larger mammals, such as humans (Drouin et al., 1998) or dogs (Litovsky and Antzelevitch, 1990). It is important to note that no matter the species, the morphology of the ventricular APs have different phases. In general, phase 0 is the upstroke of the AP, defined by the activation of voltage-gated sodium channels. Phase 1 is an early repolarization, defined by the activation of Kv 4.x K^+^ channels. This phase is typically seen in the epicardial and midmyocardial layers of mammalian ventricular myocytes. Phase 2 is the plateau phase of the AP and, arguably, one of the most important phases. During phase 2, most of the Ca^2+^ influx occurs, creating a plateau in the AP morphology. Phase 3 is the repolarization of the AP, and phase 4 is defined as the period in which the AP returns to the resting potential.

The role of the L-type Ca^2+^ current is exemplified by our data showing how the influx of Ca^2+^ through L-type Ca^2+^ channels in goldfish not only define the AP duration (duration of phase 2), but also is a key trigger in inducing Ca^2+^ release from the SR. Furthermore, we find that goldfish hearts have a similar AP temperature (Gurabi et al., 2014) and heart rate dependency (Krishnan and Antzelevitch, 1991; Burashnikov and Antzelevitch, 1998) when compared to larger mammals.

Remarkably, goldfish exhibit a negative staircase behavior in the systolic Ca^2+^ in response to an increase in heart rate, opposite to that observed in larger mammals (Langer, 1967; Opitz, 1980; Kodama et al., 1981; Bouchard and Bose, 1989; Hattori et al., 1991; Vila Petroff et al., 2003; Palomeque et al., 2004). An important component of Ca^2+^ transients during systole comes from the Ca^2+^ released from the SR. Interestingly, the fraction of Ca^2+^ released from the SR, which contributes to the amplitude of the systolic Ca^2+^ transient, is similar between goldfish and larger mammals (Belevych et al., 2007). Previous studies show how impaired Ca^2+^ release from the SR lengthens the duration of the AP (Horackova, 1986; Verduyn et al., 1995). This decrease in AP duration could imply that a Ca^2+^-dependent inactivation of the L-type Ca^2+^ channels may have an important role in controlling how much Ca^2+^ is getting into the ventricular myocytes (Bazmi and Escobar, 2019).

In conclusion, the data obtained from our experiments allow us to propose the goldfish heart as an excellent model for performing physiological experiments at the intact-heart level. Moreover, its shared similarities with larger mammals open a new avenue for goldfish hearts to be used as a model to study human physiology.

## Materials and Methods

### Heart Preparation

**FIGURE 1:**
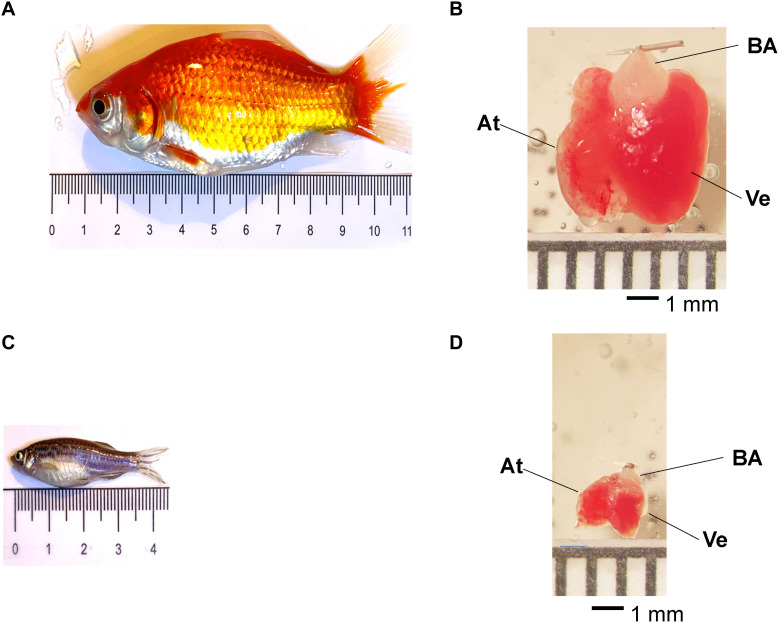
Adult goldfish measuring roughly 9.4 mm **(A)** and zebrafish measuring 3.7 mm **(C)** pictured for scale. Adult goldfish hearts **(B)** are significantly larger than adult zebrafish hearts **(D)**. It is possible to observe the ventricle (Ve), the atrium (At), and the *bulbus arteriosus* (BA) in both goldfish and zebrafish hearts.

Adult goldfish or zebrafish (Figures 1A,C) were anesthetized by immersion in ice-cold water containing 0.16 mg ml–1 tricaine methanesulfonate for 2–5 min. Zebrafish or goldfish were submerged in tanks having different volumes with respect to the fish size (Figure 1A). The tail of the fish was held with a small curved tweezer to check if each fish was completely anesthetized before decapitation. After decapitating the fish with scissors, the heart was removed from the animal’s chest. Goldfish, zebrafish, and mice were maintained in accordance with the National Institutes of Health Guide for the Care and Use of Laboratory Animals (NIH Publication No. 85–23, Revised 1996) and the Institutional Animal Care and Use Committee guidelines of the University of California Merced (Protocol # 2008–201).

### Heart Cannulation and Perfusion

Goldfish hearts (Figure 1B) were dissected and cannulated onto a 27-gauge needle and perfused in a Langendorff system at a rate of 60 μL/min driven by gravity. Multiple solutions were perfused through the *bulbus arteriosus* with the aid of a self-designed μ-manifold. Goldfish hearts were perfused with a fish ringer solution containing NaCl 137 mM, KCl 5.4 mM, CaCl_2_ 1.8 mM, MgCl_2_ 0.5 mM, HEPES 10 mM, and glucose 5.5 mM (Busselen and van Kerkhove, 1978). The Ca^2+^ dye Rhod-2 AM was perfused into the heart with a Harvard pump. The temperature of the heart was controlled with the aid of a Peltier unit positioned at the bottom of the recording chamber and measured with a linearized semiconductor temperature sensor.

Zebrafish hearts (Figure 1D) were dissected from 1-year-old fish. This was done to have larger hearts that facilitated cannulation. Next, the hearts were cannulated onto a 32-gauge needle and perfused in a Langendorff system at a rate of 10 μL/min driven by gravity through the *bulbus arteriosus*. Multiple solutions were perfused. Zebrafish hearts were perfused with a fish ringer solution containing NaHPO_4_ 0.42 mM, NaCl 150 mM, KCl 3 mM, CaCl_2_ 1.8 mM, MgCl_2_ 1.2 mM, HEPES 10 mM, and glucose 10 mM (Vornanen and Hassinen, 2016). The Ca^2+^ dye Rhod-2 AM was perfused through the zebrafish heart in an identical manner previously described for goldfish.

Mouse hearts were obtained from 8-week-old c57BL/6 male mice (Charles River Laboratories). Animals were injected intraperitoneally with Na^+^-heparin and euthanized by cervical dislocation 15 min post-injection. The heart was removed rapidly, and the aorta was cannulated onto a standard horizontal Langendorff apparatus for continuous perfusion with normal Tyrode’s solution containing, in mM, NaCl 140, KCl 5.3, CaCl_2_ 2, MgCl_2_ 1, NaPO_4_H_2_ 0.33, HEPES 10, and glucose 10. The osmolarity of the solution was 295 mOsm/L with pH 7.4. After spontaneous cardiac Ca^2+^ transients became regular, hearts were loaded with Rhod-2 with the aid of two peristaltic pumps.

### Experimental Setup

#### Optical Measurements

Ca^2+^ transients were recorded with the pulsed local field fluorescence microscopy (PLFFM) (Mejía-Alvarez et al., 2003; Aguilar-Sanchez et al., 2017). The PLFFM technique can assess physiological parameters by exciting exogenous probes present in the tissue and detecting the light emitted by these fluorescent indicators. The excitation (532 nm Yag laser) and emitted light propagate through a multimode optical fiber (200 μM diameter, 0.67 NA) placed on the surface of the intact heart. The emitted light then travels back through the multimode fiber, dichroic mirrors, and filters (610 nm) and is focused on an avalanche photodiode (Perkin Elmer, United States) with the aid of a microscope objective. The signal is digitized by an A/D converter (NI, United States) and acquired by a PC.

#### Electrophysiological Measurements

Epicardial electrical recordings of the APs were obtained using sharp glass microelectrodes filled with 3 M KCl that were connected to a high-input impedance differential amplifier (WPI, United States) in hearts paced from 1 to 3 Hz. Glass microelectrodes were fabricated with a micropipette puller (Sutter Instrument Co., United States) and had a resistance of 10–20 MΩ (Ferreiro et al., 2012; López Alarcón et al., 2019). Data were recorded with an acquisition system Digidata 1440A (Molecular Devices, Sunnyvale, CA) using pClamp 10 software. Membrane potential was always recorded from the ventricular epicardium. The hearts were paced with the aid of two acupuncture needles located in the apex of the ventricle.

#### Flash Photolysis

A flash photolysis system allows us to fractionally change a specific ionic current by photolyzing (Ramos-Franco et al., 2016). Specifically, a nifedipine partial blockade was locally relieved by UV illumination generated by a DPSS UV laser (355 nm; DPSS Lasers Inc., Santa Clara, CA). UV light was optomechanically shuttered for 1–40 ms and applied through a multimode quartz optical fiber (Escobar et al., 2012; Ramos-Franco et al., 2016; López Alarcón et al., 2019; Aguilar-Sanchez et al., 2019).

### Statistical Analysis

In whole heart experiments, there are two main causes of variance. The first cause of variance is that two different hearts cannot be completely identical to one another. The second cause of variance is that it is impossible to perform the recordings in the same precise location between multiple different hearts even though we are measuring Ca^2+^ transients and APs in the same region (the midregion of the left ventricle epicardium). Thus, the data here are presented as the measured times with their standard deviations (SD). To assess electrical changes, AP traces were evaluated at certain repolarization times. Specifically, the time it took for the AP to reach 30, 50, or 90% repolarization was assessed and is referred to as APD_30_, APD_50_, or APD_90_, respectively. To evaluate the kinetics of the recorded Ca^2+^ transients, recordings were normalized between zero (minimum fluorescence) and one (maximum fluorescence). The kinetics parameters of the Ca^2+^ transients evaluated were the rise time (RT; time for the Ca^2+^ transient to rise from 10 to 90%), half duration (HD; duration of the Ca^2+^ transient at 50% of the maximum amplitude), and fall time (FT; time for the Ca^2+^ transient to fall from 90 to 10%). Time constants τ_*on*_ and τ_*off*_ were calculated as τ_*on*_ = RT/2.2 and τ_*off*_ = FT/2.2 (Kornyeyev et al., 2012). Each recorded parameter for AP and Ca^2+^ transient kinetics, control, and non-control experiments were evaluated and normalized to the control values for each heart used. After this normalization, data were compiled, and statistical analysis was performed. The data are presented as multiple measurements (*n*; dot cloud) recorded for different measurements (*n*) on different hearts (*N*) with the mean ± SD (solid lines). In this paper, we performed 14 experiments with goldfish, 20 experiments with zebrafish, and five experiments with c57BL/6 mice. Statistical significance was tested using a two-sample Kolmogorov–Smirnov test (OriginPro 2020). The difference was significant if the *p*-value was < 0.01.

## Results

### General Properties of Goldfish and Zebrafish Excitability and Ca^2+^ Transients

Fish hearts have become an interesting model to study both cardiac physiology and pathophysiology. Specifically, zebrafish hearts have come to be a very popular model due to the possibility of developing transgenic animals. However, zebrafish present some experimental limitations when compared to goldfish. The first one is the size of the animal. Goldfish are three times longer and 2.7 times wider than the zebrafish (Figures 1A,C). Additionally, the volume of the goldfish is 20 times bigger than the zebrafish. This difference in size makes the dissection of the goldfish much easier. On the other hand, the size of the goldfish heart is also larger. For example, the goldfish ventricle is 3.7 times longer and three times wider than in the zebrafish (Figures 1B,D). Also, the total volume of the ventricle is 34 times bigger in the goldfish. Furthermore, the most important difference is the size of the *bulbus arteriosus*. The zebrafish heart needs to be cannulated using a 32- to 34-gauge needle, and the goldfish heart can be cannulated on a 27-gauge needle. The use of a larger needle for cannulation is a huge advantage because it allows for larger fluxes during perfusion and will allow for different drugs to be perfused significantly more quickly.

**FIGURE 2:**
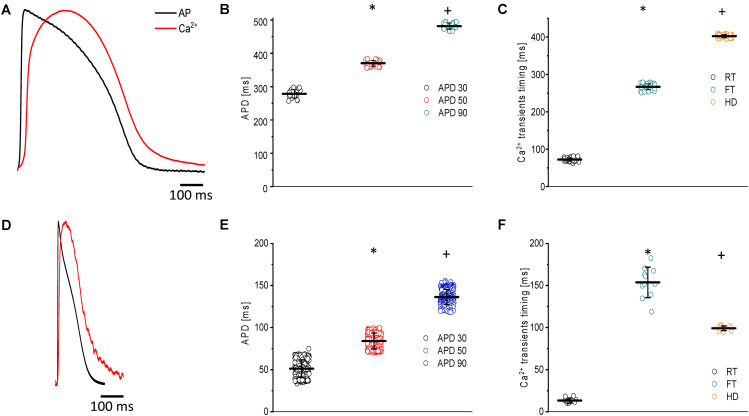
Main characteristics of both APs and Ca^2+^ transients recorded at 23°C in goldfish hearts (*N* = 14 hearts) **(A)** and zebrafish hearts (*N* = 20 hearts) **(D)**. The kinetic parameters for goldfish intact heart Ca^2+^ transients, including the rise time (72.4 ± 4.5 ms), fall time (266.9 ± 7.9 ms), and half duration (402.1 ± 4.4 ms), were established **(C)** as well as the AP durations at 30% (278.4 ± 12.4 ms), 50% (370.4 ± 8.8 ms), and 90% (481.5 ± 9.0 ms) repolarization **(B)**. The kinetic parameters for zebrafish intact heart Ca^2+^ transients, including rise time (13.3 ± 2.9 ms), fall time (153.7 ± 18.0 ms), and half duration (99.1 ± 2.7 ms), were established **(F)** as well as the AP durations at 30% (51.2 ± 10.4 ms), 50% (83.9 ± 9.4 ms), and 90% (136.5 ± 9.0 ms) repolarization **(E)**. The symbols * and + differentiate between statistically significant measurements (*p* < 0.01).

Another physiological advantage of the goldfish heart is that, at room temperature (23°C), the ventricular APs (Figures 2A,B) and Ca^2+^ transients exhibit significantly longer durations (Figures 2A,C) than those of zebrafish (Figures 2D–F). These findings make the goldfish heart a better model to be compared with larger mammals.

Figure 2A shows the main characteristics of both APs and Ca^2+^ transients recorded from goldfish. The AP parameters were longer for goldfish (Figure 2B) having an APD_30_ of 278.4 ± 12.4 ms, APD_50_ of 370.4 ± 8.8 ms, and APD_90_ of 481.5 ± 9.5 ms (*n* = 25 measurements, *N* = 14 hearts) compared to the zebrafish (Figure 2E). Specifically, zebrafish APs were observed to have an APD_30_ of 51.2 ± 10.4 ms, APD_50_ of 83.9 ± 9.4 ms, and APD_90_ of 136.5 ± 9.0 ms (*n* = 219 measurements, *N* = 20 hearts).

Several kinetic parameters, such as the RT, FT, and HD were evaluated the Ca^2+^ transients in both goldfish (Figure 2C) and zebrafish (Figure 2F). For goldfish, the kinetic parameters of the Ca^2+^ transients were *RT* = 72.4 ± 4.5 ms, *FT* = 266.9 ± 7.9 ms, and finally *HD* = 402.1 ± 4.4 ms (*n* = 40 measurements). Interestingly, the kinetic parameters for the Ca^2+^ transients were faster in zebrafish hearts (*RT* = 13.3 ± 2.9 ms, *FT* = 153.7 ± 18.0 ms, and *HD* = 99.1 ± 2.7 ms; *n* = 11 measurements).

### Role of L-type Ca^2+^ Currents in the Excitability and Ca^2+^ Transients of Goldfish Hearts

**FIGURE 3:**
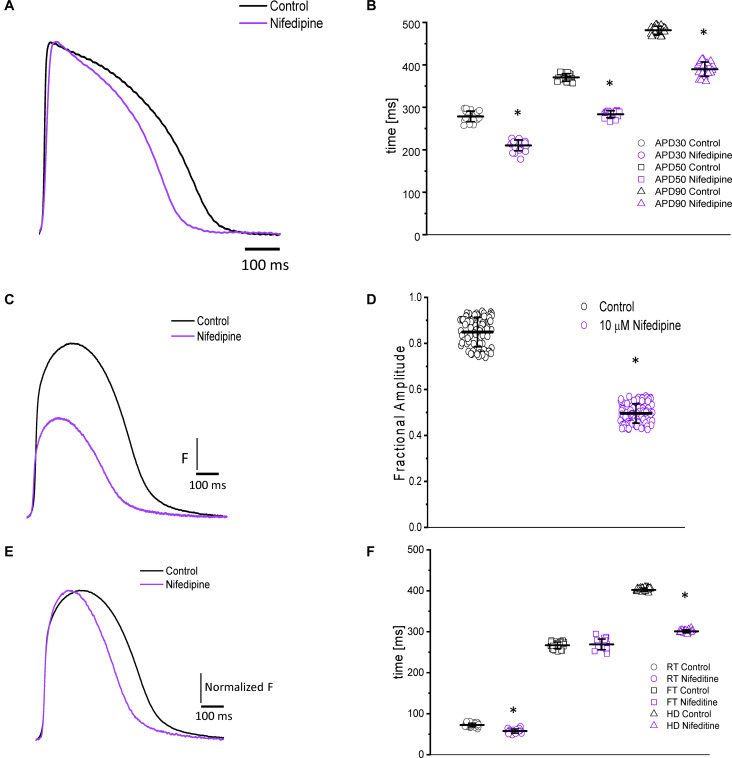
Perfusion with 10 μM nifedipine led to a significant decrease in goldfish AP duration **(A)** at 30% (278.4 ± 12.4 ms to 210.31 ± 12.84 ms), 50% (370.3 ± 8.8 ms to 283.6 ± 8.0 ms), and 90% (481.5 ± 9.5 ms to 389.5 ± 16.9 ms) repolarization **(B)**. Time course of Ca^2+^ transients in the absence and presence of nifedipine **(C)**. Perfusion with 10 μM nifedipine significantly reduced the amplitude of Ca^2+^ transient by 40% **(D)**. The normalized goldfish Ca^2+^ transient recordings **(E)** show a significant change in the kinetics of this systolic event. Furthermore, both the rise time **(F)** and half duration **(F)** of the Ca^2+^ transients were significantly reduced (from 72.4 ± 4.5 ms to 57.7 ± 5.1 ms and from 402.1 ± 4.4 ms to 300.9 ± 3.7 ms, respectively). Although the fall time of the Ca^2+^ transients increased from 266.9 ± 7.9 ms to 269.0 ± 12.9 ms, this change was not significant **(F)**. The symbol * differentiates between statistically significant measurements (*p* < 0.01, *N* = 4 hearts).

In most vertebrates, the main pathway for Ca^2 +^ influx is through the L-Type Ca^2 +^ channel. In the experiments presented in Figure 3, we address the contribution of the L-type Ca^2+^ current to the ventricular AP kinetics and the amplitude of the Ca^2+^ transients. Figure 3A illustrates the effect of partially blocking the L-type Ca^2+^ current with 10 μM nifedipine on the goldfish epicardial AP. All the parameters that define the duration of the AP decreased after the heart was perfused with nifedipine. Figure 3B shows that perfusion with nifedipine resulted in a significant reduction in the APDs. Particularly, APD_30_ was reduced from 278.4 ± 12.4 ms to 210.31 ± 12.8 ms, APD_50_ was reduced from 370.3 ± 8.8 ms to 283.6 ± 8.0 ms, and APD_90_ was reduced from 481.5 ± 9.5 ms to 389.5 ± 16.9 ms (*n* = 25 measurements, *N* = 4 hearts). These results indicate that the L-type Ca^2 +^ channel is partially responsible for the duration of the AP phase 2 (plateau phase).

Nifedipine also had a significant effect on the amplitude and the kinetics of intracellular Ca^2+^ transients. The perfusion of the goldfish hearts with 10 μM nifedipine reduced the amplitude of Ca^2+^ transient by 40% (Figures 3C,D). The amplitude of the Ca^2+^ transient was reduced from 0.84 ± 0.06 (*n* = 84 measurements) to 0.49 ± 0.04 (*n* = 106 measurements, *N* = 4 hearts). To compare the kinetics of the Ca^2+^ transients before and after nifedipine perfusion, the traces were normalized to its respected maximum amplitude (Figure 3E). The perfusion with nifedipine reduced the RT of the Ca^2+^ transients (Figure 3F) from 72.4 ± 4.5 ms (*n* = 40 measurements, *N* = 4 hearts) to 57.7 ± 5.1 ms (*n* = 22 measurements, *N* = 4 hearts). This reduction is a factor that can define the RT and the time-to-peak of Ca^2+^ transients. Consistent with the reduction in the duration of the AP, the HD of the Ca^2+^ transient was also significantly reduced (Figure 3F). Interestingly, Figure 3F shows that nifedipine perfusion reduced the RT and HD while not modifying the FT of Ca^2+^ transients. In order to assess the kinetic changes of the Ca^2+^ transient, we normalized the Ca^2+^ transients before and after perfusions with nifedipine. The normalized Ca^2+^ transient FT values before perfusion with nifedipine was 266.9 ± 7.9 ms for the control (*n* = 40 measurements, *N* = 4 hearts) and 269.0 ± 12.9 ms for hearts perfused with nifedipine (*n* = 22 measurements, *N* = 4 hearts).

**FIGURE 4:**
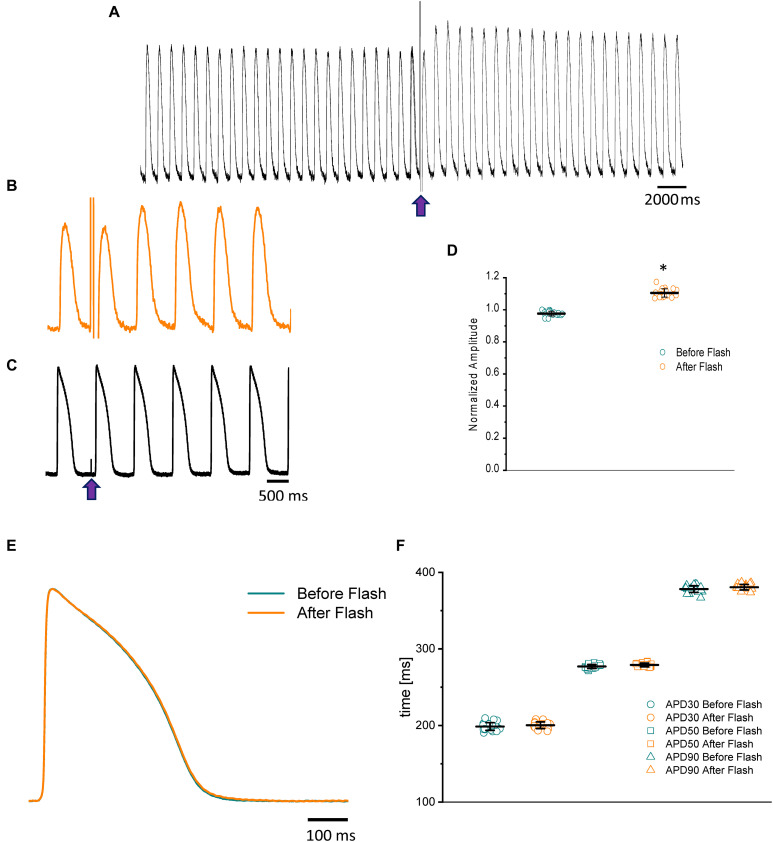
The time course of the Ca^2+^ transient recorded from goldfish hearts externally paced at 1 Hz before and after the photolytic stimulus. Simultaneous recordings of Ca^2+^ transients and AP upon introduction of a photolytic stimulus **(A,B)** reveal a significant increase in the amplitude of the Ca^2+^ transients without a measurable effect on the kinetics of the AP **(C)**. The fractional amplitude of the Ca^2+^ transient increased from 0.97 ± 0.01 before the flash to 1.11 ± 0.02 after the flash **(D)**. There was no significant change in the AP duration at 30, 50, and 90% repolarization before and after the photolytic stimulus due to the electrotonus imposed by the ventricular tissue **(E,F)**. The symbol * differentiates between statistically significant measurements (*p* < 0.01, *N* = 4 hearts).

Nifedipine likely mediated the attenuation of the Ca^2+^ transient amplitude in one of two ways. Either nifedipine reduced the amplitude of the Ca^2+^ current entering through the L-type Ca^2+^ channels, or nifedipine reduced the duration of the AP and, as such, shortened the duration of the L-type Ca^2+^ current. To discriminate between these two hypotheses, we performed experiments in which we first perfused the heart with nifedipine and then used flash photolysis to locally inactivate it. We previously demonstrated (Ramos-Franco et al., 2016) that local photolysis of nifedipine can produce a significant change in Ca^2+^ signaling without affecting the time course of the APs. This occurs because the intact heart presents important electrotonic behavior. When there is a change in the local Ca^2+^ current, the rest of the tissue will “voltage-clamp” the photolyzed volume and, thus, impede the change in the membrane potential. Figure 4 illustrates the results of the nifedipine photolysis. Figure 4A shows the time course of the Ca^2+^ transients from a heart perfused with 10 μM nifedipine and externally paced at 1 Hz before and after the photolytic stimulus. Upon photolysis of nifedipine (after the violet arrow), there was an increase in the amplitude of goldfish epicardial Ca^2+^ transients. Figure 4B and C shows simultaneous recordings of Ca^2+^ transients and AP upon a photolytic stimulus. Although there was a significant increase in the amplitude of the Ca^2+^ transients, there was not a measurable effect on the kinetics of the AP. Figures 4E,F shows that the time course of the AP does not change before and after the photolytic stimulus (*n* = 20 measurements, *N* = 4 hearts). Figure 4D illustrates the fractional increase in the amplitude of the Ca^2+^ transients after the photolytic stimulus. The fractional amplitude of the Ca^2+^ transient increased from 0.97 ± 0.01 before the flash to 1.11 ± 0.02 after the flash (*n* = 22 measurements, *N* = 4 hearts).

### Temperature Dependency of APs and Ca^2+^ Transients in Goldfish Hearts

**FIGURE 5:**
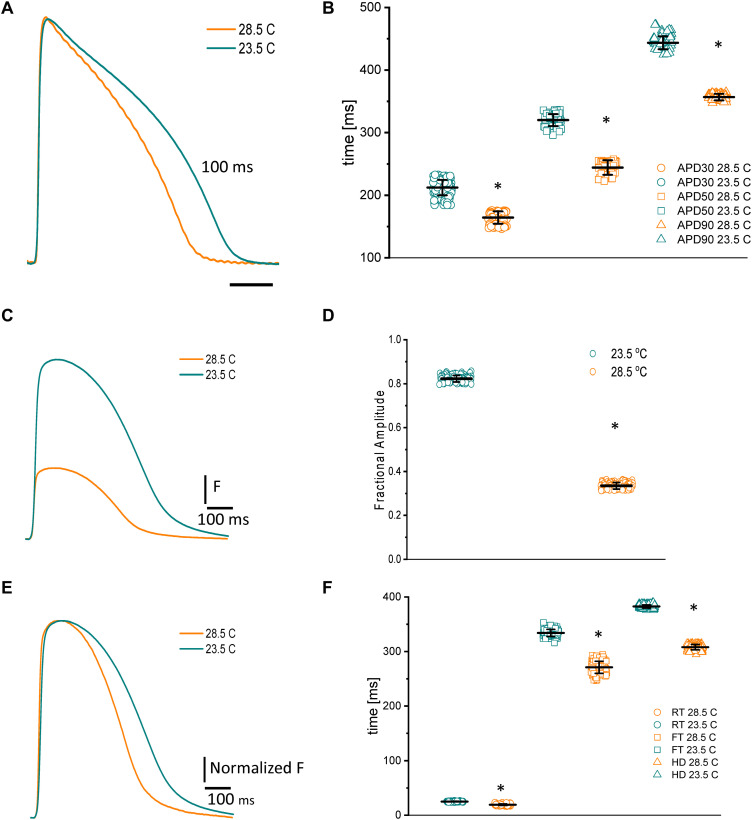
Effects of increasing the temperature from 23.5 to 28.5°C on AP and Ca^2+^ transients from goldfish hearts **(A)**. The increased temperature shortened the AP duration at 30% **(B)** (from 212.4 ± 12.3 ms to 164.3 ± 9.9 ms), 50% (from 320.1 ± 9.5 ms to 244.1 ± 11.5 ms), and 90% repolarization (from 443.5 ± 10.2 ms to 356.6 ± 5.25 ms). The increase in temperature also leads to shorter and smaller Ca^2+^ transients **(C)**. The effect of temperature on the amplitude of the Ca^2+^ transients is illustrated in panel **(D)**. Moreover, the Ca^2+^ transient traces were normalized to better observe the changes in their kinetics **(E)**. The global increase in temperature of the heart reduced the rise time **(F)** (from 25.0 ± 0.6 ms at 23.5°C to 19.25 ± 1.47 ms at 28.5°C), fall time (from 334.1 ± 6.5 ms at 23.5°C to 271.0 ± 11.1 ms at 28.5°C), and half duration (from 382.6 ± 2.9 ms at 23.5°C to 308.0 ± 4.8 ms at 28.5°C) of Ca^2+^ transients. The symbol * differentiates between statistically significant measurements (*p* < 0.01, *N* = 9 hearts).

Because fish are poikilotherm vertebrates, we decided to assess how temperature could modify both the kinetics of APs and Ca^2+^ transients. Not surprisingly, an increase in the temperature produced a shortening of the AP duration at 30, 50, and 90% repolarization, opposite that observed in mice (Ferreiro et al., 2012). Figure 5A illustrates how increasing the temperature from 23.5 to 28.5°C affects the AP. Specifically, when the temperature was increased by 5°C, the APD_30_ (Figure 5B) shortened from 212.4 ± 12.3 ms to 164.3 ± 9.9 ms, the APD_50_ (Figure 5B) shortened from 320.1 ± 9.5 ms to 244.1 ± 11.5 ms, and the APD_90_ (Figure 5B) shortened from 443.5 ± 10.2 ms to 356.6 ± 5.25 ms (*n* = 87 measurements, *N* = 9 hearts). To evaluate the thermodynamics of the AP in greater detail, we decided to calculate the first derivative to obtain the rates of depolarization and repolarization of the AP recordings at both experimental temperatures.

$$R=\frac{d\,V_{m}\,\left(t\right)}{d\,t}.$$

These rates were used to calculate the *Q*_10_.

$$Q_{10}={\left(\frac{R_{2}}{R_{1}}\right)}^{\left(\frac{10}{T_{2}-T_{1}}\right)},$$

where *R*_2_ is the rate at temperature 2, *R*_1_ is the rate at temperature 1, *T*_2_ is temperature 2, and *T*_1_ is temperature 1. The calculated *Q*_10_ for the depolarization of the AP was 1.09 and 1.56 for the repolarization of the AP.

Usually, Ca^2+^ transients have a larger temperature dependency than excitability (Kornyeyev et al., 2010; Ferreiro et al., 2012). One reason behind this increase is the temperature-dependent process of hydrolyzing ATP to pump Ca^2+^ back into the SR. To better examine this process in the goldfish, we evaluated the temperature dependency of the Ca^2+^ transients. Figure 5E exemplifies the effect of raising the goldfish heart temperature by 5°C. Upon this increase in temperature, the Ca^2+^ transients were not only smaller (Figures 5C,D), they were also faster (Figures 5E,F). The amplitude of the Ca^2+^ transients decreased from 0.82 ± 0.01 (*n* = 107 measurements) to 0.33 ± 0.01 (*n* = 107 measurements, *N* = 9 hearts). The differences in the kinetics can be better observed in Figure 5G, where the Ca^2+^ transient traces were normalized. Interestingly, the Ca^2+^ transients illustrated in Figure 5E display a smaller amplitude. However, this can be explained by the shortened AP durations at higher temperatures. Indeed, that is what we observed here. The RT (Figure 5F) decreased from 25.0 ± 0.6 ms at 23.5°C to 19.25 ± 1.47 ms at 28.5°C (*n* = 110 measurements at 23°C and *n* = 119 measurements at 28.5°C). The FT (Figure 5F) was reduced from 334.1 ± 6.5 ms at 23.5°C to 271.0 ± 11.1 ms at 28.5°C. Finally, the HD of Ca^2+^ transients (Figure 5F) diminished from 382.6 ± 2.9 ms at 23.5°C to 308.0 ± 4.8 ms at 28.5°C (*n* = 110 measurements at 23°C and *n* = 120 measurements at 28.5°C, *N* = 9 hearts). Once again, to have a better thermodynamic picture of the effect of temperature on Ca^2+^ transients we calculated *Q*_10_.

For these experiments, we first calculated the time constant for activation (*τ_*on*_*) and the time constant for the relaxation (*τ_*off*_*) of the Ca^2+^ transients. *τ_*on*_* and *τ_*off*_* were calculated as

$$\tau_{o\,n}=\frac{R\,T}{2.2}\;\mathrm{and}\;\tau_{o\,f\,f}=\frac{F\,T}{2.2}.$$

And the rates *R*_*on*_ and *R*_*off*_ were calculated as

$$R_{o\,n}=\frac{1}{\tau_{o\,n}}\;\mathrm{and}\;R_{o\,f\,f}=\frac{1}{\tau_{o\,f\,f}}$$

Then, the *Q*_10_ calculated for the Ca^2+^ transient activation was 1.68 and the relaxation of the Ca^2+^ transient was *Q*_10_ was 1.52.

### Heart Rate Dependency of APs and Ca^2+^ Transients

**FIGURE 6:**
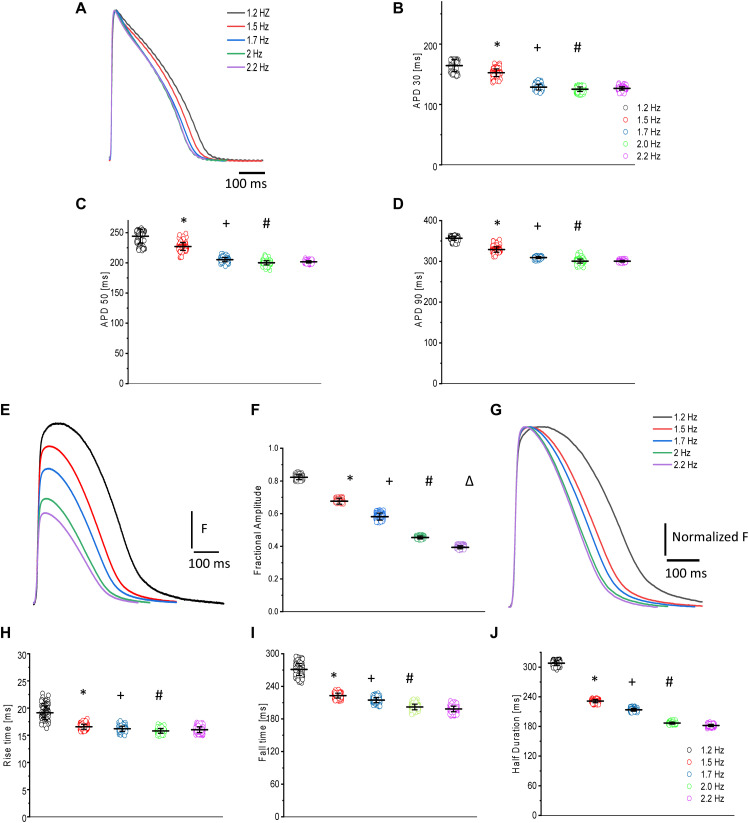
Time courses of APs and Ca^2+^ transients in goldfish hearts as a function of the heart rate **(A)**. As the heart rate increased, the duration of the APs at 30% **(B)**, 50% **(C)**, and 90% **(D)** repolarization decreased (see Table 1). The amplitude of the Ca^2+^ transients decreased **(E)** in a step staircase manner as a function of the heart rate **(F)**. There was a strong change in the kinetic behavior, which is better observed after normalization of the Ca^2+^ transient amplitude **(G)**. There was a reduction in the rise time **(H)**, fall time **(I)**, and the half duration **(J)** of the Ca^2+^ transients as a function of heart rate (see Table 2). The symbols *, +, #, and Δ differentiate measurements that are statistically significant between the two consecutive heart rates (*p* < 0.01, *N* = 6 hearts).

Like with other vertebrates, the fish heart needs to increase its heart rate to cope with environmental and stress conditions. For example, a fish escaping from a predator needs to increase the rate of skeletal muscle action potentials and contractions (Rome et al., 1993) in order to escape the predator. Here, we carried out experiments in goldfish to evaluate how an increased heart rate could modify the APs and Ca^2+^ transients (Figure 6). Figure 6A illustrates the time courses of APs as a function of the heart rate. It is important to note that as the heart rate increased, the duration of the APs got shorter. Figures 6B–D reveals how the APD_30_, APD_50_, and APD_90_ changed as a function of the heart rate, respectively (*N* = 6 hearts). A summary of the results is presented in Table 1.

**TABLE 1:** Kinetic parameters of AP as function of the heart rate.

Heart rate	1.2 Hz *n* = 87 *N* = 5	1.5 Hz *n* = 125 *N* = 5	1.7 Hz *n* = 122 *N* = 5	2.0 Hz *n* = 191 *N* = 5	2.2 Hz *n* = 184 *N* = 5
**APD**					
APD_30_ (ms)	164.3 ± 9.9	152.6 ± 6.3	128.6 ± 4.7	125.3 ± 3.7	126.5 ± 2.7
APD_50_ (ms)	244.1 ± 11.5	227.0 ± 6.4	205.3 ± 3.6	200.1 ± 3.4	201.6 ± 1.9
APD_90_ (ms)	356.6 ± 5.2	329.0 ± 6.5	309.3 ± 2.3	300.5 ± 4.9	300.3 ± 2.0

Ca^2+^ transients from the goldfish heart also present a strong heart-rate dependency. Figure 6E shows how the amplitude of the Ca^2+^ transients decreased as a function of the heart rate (Figure 6F). The traces of Ca^2+^ transients shown in Figure 6E were normalized by their maximum amplitude (Figure 6G) to gain a clearer understanding of how a change in heart rate affected Ca^2+^ transient kinetics. The Ca^2+^ transients exhibited a strong change in kinetic behavior. Specifically, as the heart rate increased, all the kinetic parameters became shorter. Figures 6H–J all depict how the amplitude, RT, FT, and HD of the Ca^2+^ transients became smaller as a function of the heart rate, respectively. An outline of the results is shown in Table 2.

**TABLE 2:** Kinetic parameters of Ca^2 +^ transients as function of the heart rate.

Heart rate	1.2 Hz *n* = 107 *N* = 5	1.5 Hz *n* = 150 *N* = 5	1.7 Hz *n* = 165 *N* = 5	2.0 Hz *n* = 200 *N* = 5	2.2 Hz *n* = 217 *N* = 5
**Times**					
Fractional amplitude	0.82 ± 0.01	0.67 ± 0.02	0.58 ± 0.02	0.45 ± 0.01	0.38 ± 0.01
Rise time (ms)	19.1 ± 1.2	16.5 ± 0.4	16.2 ± 0.4	15.8 ± 0.4	16.0 ± 0.5
Fall time (ms)	271.0 ± 11.1	222.9 ± 4.5	214.7 ± 4.7	201.9 ± 5.2	198.3 ± 5.1
Half duration (ms)	308.0 ± 4.8	231.2 ± 3.1	213.6 ± 2.8	186.9 ± 1.9	181.9 ± 2.2

### Contribution of SR Ca^2+^ Release to Ca^2+^ Transients and the Repolarization of the AP

**FIGURE 7:**
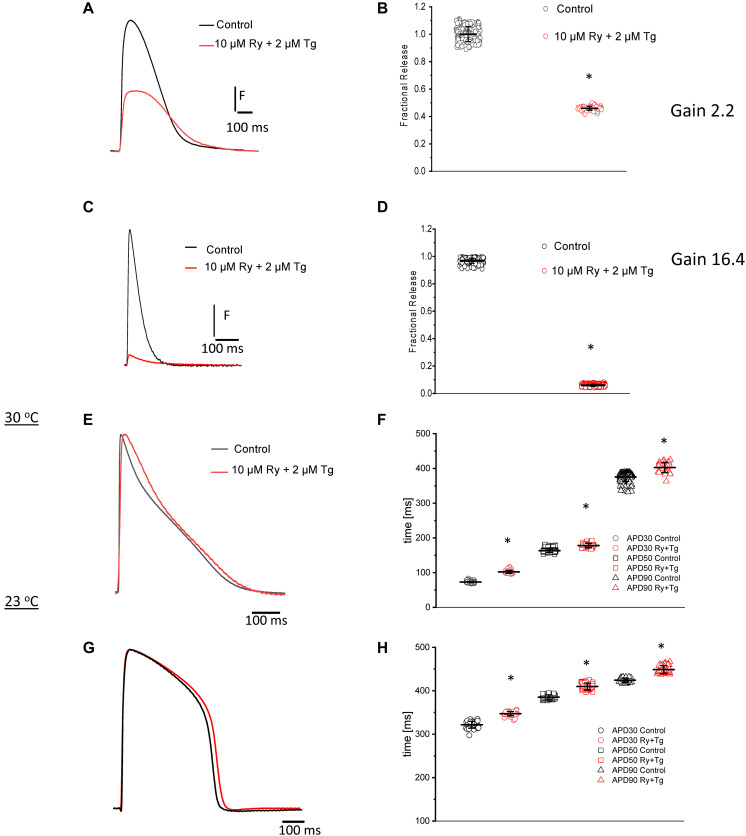
Typical Ca^2+^ transient recordings before and after perfusion of the goldfish heart with 10 μM Ry and 2 μM Tg **(A)**. A summary of the results illustrates a statistically significant decrease in the amplitude of the Ca^2+^ transient as well as a 55% decrease in the fractional release (**B**; from 1.0 ± 0.05 for the control condition to 0.45 ± 0.01 for hearts perfused with Ry and Tg). Ca^2+^ transient recordings before and after perfusion of the mouse heart with 10 μM Ry and 2 μM Tg **(C)**. Treatment with Ry and Tg in mouse hearts dramatically reduced the amplitude of Ca^2+^ transients **(D)**. The fractional release was also reduced by approximately 90% (from 0.96 ± 0.02 for control to 0.06 ± 0.007 for hearts perfused with Ry and Tg). The calculated *Gain*_*CICR*_ was 16.27 ± 0.027 for the mouse heart. Perfusion with 10 μM Ry and 2 μM Tg at 30°C impaired Ca^2+^ release from the SR **(E)** and prolonged the duration of the goldfish ventricular AP at 30% **(F)** (from 72.9 ± 2.0 ms for the control condition to 102.1 ± 4.5 ms in the presence of Ry and Tg), 50% (from 163.2 ± 5.1 ms to 177.9 ± 6.5 ms), and 90% repolarization (from 375.5 ± 12.9 ms to 406.9 ± 19.5 ms). Perfusion with 10 μM Ry and 2 μM Tg at 23°C also impaired Ca^2+^ release from the SR **(G)** and prolonged the duration of the action potential at 30% **(H)** (from 321.5 ± 7.9 ms to 347.1 ± 4.9 ms), 50% (increased from 385.53 ± 5.0 ms to 409.8 ± 7.9 ms), and 90% repolarization (from 424.3 ± 4.8 ms to 448.8 ± 8.9 ms). The symbol * differentiates measurements that are statistically significant between the two consecutive heart rates (*p* < 0.01, *N* = 5 hearts).

For numerous vertebrates, including mammals and birds, the contribution of the SR Ca^2+^ release to the total change in free cytoplasmic Ca^2+^ concentration during systole plays an essential role in determining the behavior of its Ca^2+^ transients. In order to investigate this process in the goldfish heart, we performed experiments in which we abolished the Ca^2+^ release from the SR. By perfusing the heart with 10 μM Ry and 2 μM Tg, we were able to lock the ryanodine receptor (RyRs) in a subconductance state and block the SERCA pump, respectively (Figure 7A). These effects led to a depletion in the intra-SR Ca^2+^concentration, reducing the amplitude of the Ca^2+^ transients. A summary of the results presented in Figure 7B evinces that perfusion with Ry and Tg significantly decreased the amplitude of Ca^2+^ transients (30°C) and decreased the fractional SR Ca^2+^ release from 1.0 ± 0.05 for the control condition (*n* = 204 measurements, *N* = 5 hearts) to 0.45 ± 0.01 (*n* = 60 measurements, *N* = 5 hearts), a decrease of approximately 55%. Because the Ca^2+^ transient recorded in the presence of Ry and Tg mostly represents the influx of Ca^2+^ across the plasma membrane, it is possible to calculate the gain of the Ca^2+^ induced Ca^2+^ release as

$$G\,a\,i\,n_{C\,I\,C\,R}=\frac{A\,m\,p\,l\,i\,t\,u\,d\,e_{c\,o\,n\,t\,r\,o\,l}\,o\,f\,C\,a^{2+}}{A\,m\,p\,l\,i\,t\,u\,d\,e_{R\,y-T\,g}\,o\,f\,C\,a^{2+}}.$$

The *Gain*_*CICR*_ in the goldfish heart was calculated to be 2.25 ± 0.06. Not surprisingly, mice are also a common model used to study cardiac excitability and Ca^2+^ transients’ behavior, and this inspired us to compare the goldfish model to the mice model. Similar experiments were performed in mice in order to see how the mice model compared to the goldfish model in terms of SR Ca^2+^ release contribution. The amplitude of Ca^2+^ transient dramatically decreased upon perfusion with Ry and Tg (Figure 7C), and this trend was consistent across multiple Ca^2+^ transient measurements (Figure 7D). The fractional release also decreased from 0.96 ± 0.02 for the control condition (*n* = 239 measurements, *N* = 5 hearts) to 0.06 ± 0.007 for hearts perfused with Ry and Tg (*n* = 626 measurements, *N* = 5 hearts), a decrease of 90% (Figure 7D). Finally, the *Gain*_*CICR*_ was calculated to be 16.27 ± 0.027 for the mouse heart.

Ca^2+^ released from the SR not only alters the Ca^2+^ transient, but it can also have a drastic impact on the repolarization of the AP. Experiments presented in Figures 7E,F were designed to discern how Ca^2+^ release from the SR impacted AP repolarization. Figure 7E indicates that impairment of Ca^2+^ release from the SR, induced by Ry and Tg, prolonged the duration of the goldfish ventricular AP at 30°C. APD_30_ displayed an increase (Figure 7F) from 72.9 ± 2.0 ms for the control condition (*n* = 180 measurements, *N* = 5 hearts) to 102.1 ± 4.5 ms in the presence of Ry and Tg (*n* = 33 measurements, *N* = 5 hearts). Following a similar trend, perfusion with Ry and Tg prolonged APD_50_ (Figure 7F) from 163.2 ± 5.1 ms (*n* = 180 measurements, *N* = 5 hearts) to 177.9 ± 6.5 ms (*n* = 33 measurements, *N* = 5 hearts) and APD_90_ (Figure 7F) from 375.5 ± 12.9 ms (*n* = 180 measurements) to 406.9 ± 19.5 ms (*n* = 33 measurements).

The prolongation of the AP duration by Ry and Tg was also observed in goldfish ventricular APs recorded at 23°C. Figure 7G shows that, although the APs are longer at this lower temperature, Ry and Tg still prolonged the AP duration at all the levels. APD_30_ changed (Figure 7H) from 321.5 ± 7.9 ms (*n* = 40 measurements) to 347.1 ± 4.9 ms (*n* = 40 measurements, *N* = 5 hearts), APD_50_ (Figure 7H) increased from 385.53 ± 5.0 ms (*n* = 40 measurements) to 409.8 ± 7.9 ms (*n* = 40 measurements), and APD_90_ (Figure 7H) increased from 424.3 ± 4.8 ms (*n* = 40 measurements, *N* = 5 hearts) to 448.8 ± 8.9 ms (*n* = 40 measurements, *N* = 5 hearts). These results suggest that Ca^2+^-dependent inactivation of the L-type Ca^2+^ channel is the most likely mechanism prolonging the AP duration in the absence of Ca^2+^ release from the SR.

## Discussion

### Comparison Between Goldfish Hearts and Other Vertebrates

Goldfish (*Carassius auratus*) are members of the same phylum (*Chordata*), class (*Actinopterygii*), order (*Cypriniformes*), and family (*Cyprinidae*) as the zebrafish (*Danio rerio*). Both species can also regenerate their heart following injury (Poss et al., 2002; Grivas et al., 2014; Kang et al., 2016; Mokalled et al., 2016). Though the similarities between the goldfish and the zebrafish can form an extensive list, there are a few exigent differences between the two models. The most obvious difference being the adult goldfish (Figures 1A,C) and its heart (Figures 1B,D) are both significantly larger when compared to the zebrafish. It is important to note that goldfish and zebrafish ventricular myocytes do not present t-tubules, contrary to mammalian ventricular myocytes. Though this structural difference may seem significant, we do not expect the absence of t-tubules to dramatically affect the time course of Ca^2+^ transients. Indeed, numerous animal models lack a t-tubule network. For example, mammalian atrial cells have a highly reduced t-tubules network (McNutt and Fawcett, 1969; Michailova et al., 2002; Richards et al., 2011), which is compensated for by the presence of surface dyads and a Ca^2+^-induced Ca^2+^ release and Ca^2+^ propagation within the myocytes (Hüser et al., 1996). Furthermore, ventricular myocytes in birds also lack a t-tubule network (Sheard et al., 2019), and yet they present fast Ca^2+^ transients. The important parameter critical in defining the time course of the Ca^2+^ transients is not the presence (or absence) of a t-tubule network, but the time course of the Ca^2+^ influx across the plasma membrane and the kinetics of activation of ryanodine receptors.

A noteworthy difference between the zebrafish and goldfish models is that the heart rate for goldfish (109 beats/min) (Ferreira et al., 2014) is much closer to larger mammals (i.e., canines and humans) when compared to the zebrafish heart rate (162–169 beats/min) (Lee et al., 2016). Furthermore, Figure 2A describes both the characteristics of APs and Ca^2+^ transients recorded in goldfish ventricles. The goldfish AP is significantly longer than that of zebrafish (Figures 2B,E). Interestingly, the APD_90_ recorded in goldfish ventricular myocytes (Figure 2B) is very similar to values recorded in the endocardial ventricular layer of dog hearts (Piktel et al., 2011) at a similar temperature (25°C). Additionally, the epicardial APs in goldfish present a waveform very similar to the one recorded at the dog endocardial layer, in that the goldfish epicardial AP do not present a “spike and dome” behavior when compared to epicardial AP recorded from dogs (Litovsky and Antzelevitch, 1990; Antzelevitch et al., 1991; Lukas and Antzelevitch, 1993; Yan and Antzelevitch, 1996). Finally, the goldfish APD_90_ is very similar to the human QT segment at the same temperature (Bjørnstad et al., 1994).

The kinetic parameters (i.e., RT, FT, and HD) for goldfish Ca^2+^ transients (Figures 2A,C) are much slower, not only when compared to the zebrafish (Figures 2D,F), but also when compared to rodents, such as rats (Zoghbi et al., 2000, 2004) and mice (Escobar et al., 2006, 2012; Ferreiro et al., 2012). Interestingly, the goldfish Ca^2+^ transients present similar kinetic characteristics within canine hearts both in isolated myocytes (A. E. Belevych et al., 2011) and at the intact heart level (Laurita et al., 2003).

### Role of the L-type Ca^2+^ Channels in Excitability and Ca^2+^ Transients of the Goldfish Heart

In most vertebrate species, the influx of Ca^2+^ through the L-type Ca^2+^ channel is critical in defining the time course of APs and is the main mechanism triggering ventricular Ca^2+^ release. Results presented in Figures 3, 4 depict the role of L-type Ca^2+^ currents in goldfish excitability and its ability to activate the excitation–contraction coupling process.

Previous studies conducted on fish hearts show how nifedipine perfusion can block L-type Ca^2+^ currents (Maylie and Morad, 1995) and reduce the duration of the AP (Nemtsas et al., 2010); this is nearly identical to what has been previously observed in mammals (Sugiura and Joyner, 1992; Baláti et al., 1998; Ferreiro et al., 2012; Ramos-Franco et al., 2016). Not surprisingly, the same pattern was also observed in goldfish hearts (Figures 3A,D), suggesting there is an influx of Ca^2+^ through L-type Ca^2+^ channels that occurs during the plateau phase of the ventricular AP.

One consequence of a decreased Ca^2+^ influx during the plateau phase of the AP is a reduction in the amplitude of the Ca^2+^ transients. Upon perfusion of the goldfish heart with nifedipine, we observed an attenuation of the Ca^2+^ transient during systole as well as a change in the kinetic behavior of the Ca^2+^ transients (Figures 3C–F). It has been previously reported that nifedipine reduces the amplitude of Ca^2+^ transients in some fish models (Xie et al., 2008; Bovo et al., 2013; van Opbergen et al., 2018a); however, there is no evidence of Ca^2+^ transient shortening. This is interesting because several mammalian models exhibit a shortening in the HD of the Ca^2+^ transients in response to nifedipine (Escobar et al., 2004; Ramos-Franco et al., 2016).

As we describe in the “Results” section, the amplitude of the Ca^2+^ transient can decrease as a result of a smaller L-type Ca^2+^ current amplitude. Additionally, a reduction in the amplitude of the Ca^2+^ transient can be induced by shortening the AP. This induces a shortening of the L-type Ca^2+^ current and brings fewer Ca^2+^ ions into the ventricular myocyte. In goldfish, we observed a nifedipine-driven change in the amplitude of the Ca^2+^ transient that was independent of the AP duration (Figure 4). In Figure 4, we demonstrate that the photolytic inhibition of a fraction of nifedipine blocking the L-type Ca^2+^ channels produced an increase in the amplitude of the Ca^2+^ transient (Figures 4A–D) without a change in the kinetic properties of the AP (Figures 4E,F). This is analogous to what our group previously observed in experiments conducted in mouse hearts (Ramos-Franco et al., 2016).

### Temperature Dependency of APs and Ca^2+^ Transients

Fish cannot systemically regulate their body temperature; thus, the temperature of the water has a large influence on the physiological behavior of the animal’s cardiac function (Ferreira et al., 2014; Vornanen, 2016). We illustrate this idea in Figure 5, in which both the time course of the AP and Ca^2+^ transients of goldfish hearts display a significant temperature dependency. Interestingly, the depolarization of the AP has a much lower temperature dependency (*Q*_10_) than the repolarization (Figures 5A,B). The depolarization of an AP is mainly dependent on the activation of voltage-dependent Na^+^ channels, thus expounding the observed thermodynamic behavior. The repolarization, on the other hand, can include multiple events that can be regulated by the Ca^2+^ released from the SR. Some mechanisms regulating the repolarization of the AP include the activation of the Na^+^–Ca^2+^ exchanger in its forward mode and/or the Ca^2+^-dependent inactivation of the L-type Ca^2+^ channel. The reduction in the AP duration as a function of the temperature increase has been reported by other authors in other fish models (Lin et al., 2014; Vornanen, 2016; Badr et al., 2018; Rayani et al., 2018). However, there was no information about the Ca^2+^ transients’ amplitude and its temperature dependency in fish models.

Remarkably, the amplitude (Figures 5C,D), activation (Figures 5E,F), relaxation (Figure 5F), and HD (Figure 5F) of goldfish ventricular Ca^2+^ transients also present a significant temperature dependency (Figures 5C,E). Interestingly, raising the temperature by only 5°C significantly reduced the amplitude of the Ca^2+^ transient (Figures 5C,D). Moreover, the decrease in the Ca^2+^ transient amplitude can be due to the shortening of the AP at higher temperatures. If the AP is shorter, then the duration of the L-type Ca^2+^ current is also shorter and less Ca^2+^ is brought into the cell. A very similar temperature dependency has been reported in other species (Edman et al., 1974; Mattiazzi and Nilsson, 1976), in which the mechanical activity and the force–velocity relationship were measured in rabbit papillary muscle. The authors conclude that the observed decrease in contractility cannot be solely related to a decrease in the duration of Ca^2+^ release, but may also be related to an increase in the rate of Ca^2+^ sequestration by the SR.

The temperature dependency of the activation and relaxation of the Ca^2+^ transients presents several interesting features. The RT and the activation time constant of the Ca^2+^ transient have a larger temperature dependency (*Q*_10_) than the FT and the relaxation time constant. This thermodynamic behavior can be attributed to the highly cooperative process of Ca^2+^ release from the SR, which depends on the intra-SR Ca^2+^ content (Kornyeyev et al., 2010). Furthermore, as the Ca^2+^ content depends on the activity of the SERCA pump, and this pump needs to hydrolyze ATP to reload the SR, it is expected that this process will have a substantial temperature dependency. On the other hand, the relaxation of the Ca^2+^ transient depends on multiple factors, such as the binding of Ca^2+^ to intracellular Ca^2+^ buffers, which usually has a weak temperature dependency. Altogether, it is logical to expect that relaxation will have a smaller temperature dependency than the activation of the Ca^2+^ transients (Genge et al., 2016; Rayani et al., 2018).

### Heart Rate Dependency of APs and Ca^2+^ Transients

The AP duration of goldfish hearts changes significantly in response to a change in heart rate, suggesting AP duration to be frequency dependent (Figures 6A–D). The profile of decrease of the AP duration at the three levels (i.e., APD_30_, APD_50_, and APD_90_), shows a similar profile to those previously observed for mammals (Litovsky and Antzelevitch, 1989; Lukas and Antzelevitch, 1993; Ferreiro et al., 2012) and other fish models (Lin et al., 2014, 2015; Rayani et al., 2018).

Another important outcome of the heart rate dependency in goldfish hearts is the effect on the Ca^2+^ transient amplitude (Figure 6E). Specifically, the appearance of a negative staircase behavior emerges in response to an increased heart rate (Figure 6F). All the kinetic parameters, such as the RT, FT, and HD of the Ca^2+^ transients became faster (Figures 6G–J) as observed in other fish models (Lin et al., 2015; Rayani et al., 2018). In terms of the negative staircase behavior of Ca^2+^ transients, there are reports for other fish models related to a decrease in the amplitude of Ca^2+^ transients (Lin et al., 2015) and in developed pressure (Haustein et al., 2015). Interestingly, although mice display a significant negative staircase profile in the amplitude of the Ca^2+^ transients (Kornyeyev et al., 2012), larger mammals present a positive staircase profile in their mechanical response (Langer, 1967; Kodama et al., 1981; Opitz, 1980; Bouchard and Bose, 1989; Hattori et al., 1991). The discrepancy in the staircase behavior observed between larger mammals and goldfish could imply that goldfish not only have a faster Ca^2+^ reloading rate of the SR, but also have a more prominent Ca^2+^-dependent inactivation of L-type Ca^2+^ channels.

### Role of Intracellular Ca^2+^ Release on Excitability and Ca^2+^ Transients

Depending on the vertebrate species, the Ca^2+^ released from the SR can play either a major or minor role in contributing to the systolic Ca^2+^ during the cardiac cycle. The role of the Ca^2+^ release from the SR ranges dramatically and can be made evident when comparing frogs, in which Ca^2+^ release from the SR is not present (Anderson et al., 1989), to mice, in which most of the Ca^2+^ increase during diastole depends on Ca^2+^ released from this intracellular store (Ferreiro et al., 2012; Kornyeyev et al., 2012; Ramos-Franco et al., 2016).

Figures 7A,B shows that, in goldfish hearts, there is a significant contribution from the SR to the myoplasmic systolic Ca^2+^; however, this contribution is less than the one observed in smaller mammals such as mice (Figures 7C,D). This was especially impressive because previous experiments performed in fish-isolated myocytes only showed a very small contribution of the SR (Shiels and White, 2005; Bovo et al., 2013) to the free systolic Ca^2+^. Furthermore, the lower contribution of the SR Ca^2+^ release of goldfish hearts in comparison with the mouse is encouraging because larger mammalian models, such as dogs, also present a lower SR Ca^2+^ contribution (∼40%) (Belevych et al., 2007).

Interestingly, the inhibition of Ca^2+^ release from the SR promoted by the perfusion of the goldfish hearts with Ry and Tg also modified the repolarization of ventricular AP. Figures 7E,F illustrates that, in the presence of Ry and Tg, the APs are longer at both 30°C and 23°C (Figures 7G,H). Increased Ca^2+^-dependent inactivation of L-type Ca^2+^ channels shortens the action potentials when Ca^2+^ release is larger but reduced Ca^2+^-dependent inactivation prolongs the action potential if the Ca^2+^ release from the sarcoplasmic reticulum is impaired. Interestingly, this effect is contrary to what we observe in mice hearts. Upon coronary perfusion of mice hearts with Ry and Tg, the minuscule amount of Ca^2+^ released from the SR is unable to activate the Na^+^– Ca^2+^ exchanger in the forward mode and, thus, shortens the AP duration. On the other hand, the Na^+^–Ca^2+^ exchanger can also prolong the AP for a larger Ca^2+^ release while in the forward mode by activating an inward Na^+^ current (Ferreiro et al., 2012; Ramos-Franco et al., 2016). This is because the Na^+^–Ca^2+^ exchanger in the forward mode removes one Ca^2+^ ion from the cytosol to produce an influx of three Na^+^ ions from the extracellular space to the cytosol. Thus, a larger increase in the intracellular Ca^2+^ concentration results in a larger Ca^2+^ extrusion from the cytosol. This large extrusion, consequently, increases Na^+^ influx from the extracellular space into the cytosol. The net influx of a positive charge from the Na^+^ ions induces a depolarization of the membrane potential, prolonging the duration of the AP. Interestingly, the same AP prolongation behavior observed in goldfish hearts in response to Ry and Tg has been previously observed in dog hearts, in which BAPTA-mediated attenuation of Ca^2+^ release prolonged the AP (Horváth et al., 2016). Although the prolongation of the AP is due to the effect of SR Ca^2+^ release on Ca^2+^-dependent inactivation, changes in the time course of the action potential also change the delay-rectifying and inward-rectifying K^+^ current. Furthermore, prolongation of the AP also leads to an inactivation of the Na^+^ current, preventing alteration of Na^+^ currents in a major way.

## Conclusion

The goldfish heart presents several physiological attributes, making it a suitable choice to study physiological and pathophysiological problems presented in larger mammals, such as humans. Those characteristics include heart rate, duration of AP kinetics of Ca^2+^ transients, role of the L-type Ca^2+^ channel triggering excitation–contraction coupling, temperature and heart rate dependency, and contribution of the SR Ca^2+^ release to Ca^2+^ transients and excitability. All these factors open the window for the goldfish heart to be used in order to assess human and larger mammals’ cardiac function.

## Data Availability Statement

The raw data supporting the conclusions of this article will be made available by the authors, without undue reservation.

## Ethics Statement

The animal study was reviewed and approved by the Institutional Animal Care and Use Committee UC Merced (AUP # 17-0005).

## Author Contributions

MB and AE designed the research, performed the research, analyzed data, and wrote the manuscript. Both authors contributed to the article and approved the submitted version.

## Conflict of Interest

The authors declare that the research was conducted in the absence of any commercial or financial relationships that could be construed as a potential conflict of interest.
